# Attenuated T Cell Responses Are Associated With the Blockade of Cerebral Malaria Development by YOP1-Deficient *Plasmodium berghei* ANKA

**DOI:** 10.3389/fimmu.2021.642585

**Published:** 2021-05-06

**Authors:** Lei Hai, Xiaoyu Shi, Qian Wang

**Affiliations:** ^1^ Department of Immunology, School of Basic Medical Sciences, and Tianjin Key Laboratory of Cellular and Molecular Immunology, Key Laboratory of Immune Microenvironment and Diseases of Educational Ministry of China, Tianjin Medical University, Tianjin, China; ^2^ National Laboratory of Biomacromolecules, Institute of Biophysics, Chinese Academy of Sciences, Beijing, China

**Keywords:** YOP1, *Plasmodium berghei*, cerebral malaria, T cell, immune response

## Abstract

Reticulon and the REEP family of proteins stabilize the high curvature of endoplasmic reticulum tubules. The REEP5 homolog in *Plasmodium*, *Plasmodium berghei* YOP1 (*Pb*YOP1), plays an important role in the erythrocytic cycle of the *P. berghei* ANKA and the pathogenesis of experimental cerebral malaria (ECM), but the mechanisms are largely unknown. Here, we show that protection from ECM in *Pb*yop1Δ-infected mice is associated with reduced intracerebral Th1 accumulation, decreased expression of pro-inflammatory cytokines and chemokines, and attenuated pathologies in the brainstem, though the total number of CD4^+^ and CD8^+^ T cells sequestered in the brain are not reduced. Expression of adhesive molecules on brain endothelial cells, including ICAM-1, VCAM-1, and CD36, are decreased, particularly in the brainstem, where fatal pathology is always induced during ECM. Subsequently, CD8^+^ T cell-mediated cell apoptosis in the brain is compromised. These findings suggest that *Pb*yop1Δ parasites can be a useful tool for mechanistic investigation of cerebral malaria pathogenesis.

## Introduction

Malaria is caused by protozoan parasites of the genus *Plasmodium* and remains a leading cause of death and disease across many tropical and subtropical countries. An estimated 229 million cases of malaria and 409,000 deaths per year have been reported, mostly children under 5 years of age in sub-Saharan Africa (1). Cerebral malaria (CM) is the most severe complication of *Plasmodium falciparum* infection and a major cause of death in severe malaria. Mechanistic investigations of CM in humans are difficult for ethical reasons (2). Alternatively, an experimental cerebral malaria (ECM) model with *Plasmodium berghei* ANKA infection in C57BL/6 mice has been widely used (3). The pathological features of ECM include increased pro-inflammatory cytokines, vascular pathology, disruption of the blood-brain barrier (BBB), and cerebral edema, in a similar fashion to that reported in human CM (3–5). Therefore, the ECM model is a valuable tool to elucidate the mechanisms of CM.

The endoplasmic reticulum (ER) likely plays an important role in *Plasmodium* infection. The organelle is involved in vital cellular processes, such as protein translation and secretion, lipid biosynthesis, and calcium homeostasis (6, 7), and as such is directly linked to surface remodeling of infected red blood cells (iRBCs), which in turn regulate sequestration and host immune responses. In all eukaryotic cells, the ER forms a continuous membrane system of tubules and sheets, the shape of which is tightly associated with its physiological functions (8, 9). An initial analysis of the plasmodial ER identified three homologs of proteins that stabilize membrane curvature and generate ER tubules in *P. berghei* ANKA, termed *Pb*YOP1, *Pb*YOP1L, and *Pb*RTN1 (10). To investigate the importance of ER morphogenesis in *Plasmodium*, we generated YOP1-deficient *P. berghei* parasites (*Pb*yop1Δ) and found that the growth rate and virulence in ECM are severely attenuated during blood-stage infection (11). The decreased growth rate in *Pb*yop1Δ parasites is caused by the disordered digestive vacuole biogenesis associated with abnormal hemoglobin degradation. However, the mechanism of protection against ECM in *Pb*yop1Δ parasite-infected mice is unclear. In this study, we investigated the immune response and pathologies in the brain induced by *Pb*yop1Δ parasite infection during ECM induction. We found that T cells were efficiently trapped in the mouse brain, but Th1 cells were reduced compared to wild-type (WT) parasite-infected mice and the secretion of pro-inflammatory cytokines and chemokines largely decreased. In addition, reduced expression of necessary adhesive molecules on the endothelial cell and decreased expression of perforin and granzyme B leads to insufficient killing of intracerebral cells by CD8^+^ T cells.

## Material And Methods

### Ethics Statement

All animal work in this study was approved by the Institutional Animal Care and Use Committee (IACUC) of Tianjin Medical University (TMU), and was performed in accordance with ethical standards in the Laboratory Animal Guideline for Ethical Review of Animal Welfare (The National Standard of the People’s Republic of China GB/T 35892-2018).

### Animals and Parasites

Female C57BL/6 mice aged 6-8 weeks were purchased from SPF (Beijing) Biotechnology Co., Ltd (Beijing, China) and maintained at the Animal Care Facilities of Tianjin Medical University.

*P. berghei* ANKA lines (*clone 15Cy1*) were kindly gifted from Dr. Purnima Bhanot, Rutgers New Jersey Medical School, Newark, USA. Blood-staged *P. berghei* ANKA parasites were stored in liquid nitrogen and thawed for using in all experiments. Parasitemia was monitored by counting the number of iRBCs per 2000 total RBCs under light microscopy examination of Giemsa-stained thin smears of tail blood.

### Experimental Cerebral Malaria Construction and Assessment

Cryopreserved *P. berghei* ANKA parasite was thawed and passaged once *in vivo* before being used to infect experimental animals. C57BL/6 mice were infected *via* intravenous injection of 1×10^4^ WT parasites-infected RBCs or 1×10^4^ or 1×10^6^ *Pb*yop1Δ parasites-infected RBCs. The parasitemia of each mouse was recorded from 5 to 7 days post-infection (dpi). Mice were monitored daily for survival and neurological signs of ECM, such as ataxia, paralysis, and coma. According to Ana Villegas-Mendez et al. (12), signs of disease could be classified into five stages using the following clinical scale: 1 = no signs; 2 = ruffled fur and/or abnormal posture; 3 = lethargy; 4 = reduced responsiveness to stimulation and/or ataxia and/or respiratory distress/hyperventilation; and 5 = prostration and/or paralysis and/or convulsions. All animals were immediately euthanized when observed at stage 4 or 5.

### Mononuclear Cell Isolation

To determine the migration of CD4^+^ and CD8^+^ T cells to the brain, brain mononuclear cells were isolated from the brain of mice 7 dpi following a previously reported procedure (13). Briefly, anesthetized mice received an intracardiac perfusion with 1×PBS to remove all blood leukocytes and other non-adhered cells. Brains were dissected and chopped into small pieces and incubated in 1×HBSS with collagenase/dispase (1 mg/ml; Roche, Germany) for 30 min at 37°C. The suspension was filtered through a 70-μm cell strainer (Falcon, USA) and the volume of the cell suspension brought up to 7 ml with HBSS. We added 3 ml of 100% Percoll (GE Healthcare, Sweden) gradient to the cell suspension to achieve a final 30% gradient. The total 10 ml cell suspension was slowly overlaid on 2 ml of a 70% Percoll gradient and centrifuged at 500*g* for 30 min at 18°C with no brake. The 70%-30% interphase was gently removed to a clean tube containing 8 ml 1×HBSS, mixed a few times by interversion, and centrifuged at 500*g* for 7 min at 18°C. The pellet was collected and treated with ACK lysing buffer to remove RBCs, washed, and resuspended in flow cytometry buffer (1×PBS containing 1% FBS).

To determine the CD4^+^ and CD8^+^ T cell dynamic in peripheral blood, peripheral blood was collected by cardiac puncture and mixed with sodium heparin for anticoagulation. Peripheral blood mononuclear cells were isolated using the Mouse Peripheral Blood Mononuclear Cell Isolation Kit (Solarbio, China) according to the manufacturer’s protocol. The absolute number of mononuclear cells from the brain and peripheral blood were determined using a hemocytometer, and live cells were distinguished from dead cells using trypan blue staining.

### Flow Cytometry

The following antibodies and reagents from eBioscience or BD were used: CD3e-FITC (145-2C11), CD4-PE (RM4-5), CD4-PerCP (RM4-5), CD8-APC (53-6.7), CXCR3-PE (CXCR3-173), T-bet-PE (eBio4B10), Foxp3-PE (MF23), CD25-APC (PC61), and CD16/32 (93). Before staining, all cell preparations were incubated with anti-mouse CD16/32 (Fc receptor block) for 15 min on ice to reduce nonspecific antibody binding. For surface staining, cells were incubated with cocktails of mAbs in flow cytometry buffer. For intracellular staining, live cells were incubated with PMA (200 ng/ml; Solarbio, China) and ionomycin (1 μg/ml; Cayman Chemical, USA) in the presence of brefeldin A (1:1000; eBioscience, USA) for 5 h at 37°C in 5% CO_2_. Cell suspensions were first stained with surface antibodies, then treated with Foxp3/Transcription Factor Staining Buffer Set (eBioscience, USA) according to the manufacturer’s instructions before staining intracellularly with anti-mouse T-bet or Foxp3. The single-color controls and isotype-control Abs were used to validate the flow cytometry results. Samples were acquired using a Canto II flow cytometer (BD) and the data were analyzed using FlowJo software version 7.6.1.

### Real-Time PCR

Total RNA was extracted with TRIzol reagent (Invitrogen, CA, USA) and cDNA synthesis was performed using a reverse transcription kit (CWBIO, China) according to the manufacturer’s instructions. The mRNA level of each gene was measured using the SYBR Green PCR Master Mix (CWBIO, China) and performed on a LightCycler^®^ 96 System (Roche, Basel, Switzerland). The relative mRNA expression levels were evaluated using the 2^−ΔΔCt^ method and normalized to the housekeeping gene mouse β-actin. The level of parasites sequestered in the brain was determined as the expression of *P. berghei* ANKA 18S rRNA. The primer sequences are shown in **Supplementary Table 1**.

### ELISA

Blood was collected by retro-orbital bleeding 7 dpi. Blood samples were allowed to clot for 30 min at room temperature, and spun at 2700*g* for 10 min at 4°C. The serum was collected and analyzed for IFN-γ and TNF-α levels *via* ELISA (Lianke, China). The minimum limits of detection for IFN-γ and TNF-α are 3.9 pg/ml and 1.63 pg/ml, respectively.

### Immunohistochemistry

Mice were anesthetized with chloral hydrate and transcardially perfused with 20 ml of ice-cold 1×PBS. Brains were dissected and fixed in 4% paraformaldehyde for 24 h and processed for paraffin embedding. Sagittal brain sections (4 μm) were prepared for immunohistochemistry (IHC). After dewaxing and rehydration, brain sections were subjected to heat-mediated antigen retrieval in pre-heated sodium citrate buffer (pH 6.0) at 95°C for 30 minutes, and then allowed to cool at room temperature for at least 2 h. Slides were washed with 1× PBS, blocked with 5% goat serum in 1×PBST (with 0.1% Tween-20) for 1 h, and then incubated with anti-ICAM-1 (1:200; Santa Cruz Biotechnology, G-5), anti-VCAM-1 (1:200; Cell Signaling Technology, D2T4N), or anti-CD36 antibody (1:200; Cell Signaling Technology, D8L9T) in primary antibody dilution buffer overnight at 4°C. Next, the sections were washed three times with 1×PBST, incubated with HRP-labeled secondary antibody (ZSGB-BIO, China) for 1 h, washed three additional times, and then DAB (ZSGB-BIO, China) dropped onto the section as the substrate. Finally, the sections were counterstained with hematoxylin, washed, dehydrated, and sealed by coverslip with neutral balsam. Each field was chosen at random within one of four regions: olfactory bulb, cerebrum, brain stem, or cerebellum. The sections were visualized and the images acquired by a Nikon ECLIPSE 90i microscope using NIS-Elements BR (version 3.1) software. Positive vessels were counted in 6 (olfactory bulb), 20 (cerebrum), 15 (brain stem), or 5 fields (cerebellum) for each brain section per mouse at ×10 objective. Images are shown at ×40 objective.

### Western Blotting

Brain tissues were homogenized and lysed in RIPA buffer supplemented with protease inhibitor cocktail (Roche, Germany). After extraction, the protein concentration was determined using the BCA Protein Assay Kit (ThermoFisher, USA). Approximately 50 μg of protein was used for SDS-PAGE and transferred to a PVDF membrane (Roche, Basel, Switzerland). Proteins were probed with anti-Caspase3 (1:1000; 9662S, Cell Signaling Technology, USA) or anti-β-tubulin antibody (1:1000; 2146S, Cell Signaling Technology, USA). Antibody binding was revealed using an HRP-conjugated goat anti-rabbit IgG (H+L) (1:3000; Sungene Biotech, China). Antibody complexes were detected using Immobilon Western HRP Substrate (Millipore, Germany) and exposed on a Tanon-5200 machine.

### Apoptosis Detection *In Situ*

The sagittal brain sections were prepared as described above for IHC. Apoptotic cells were detected *in situ* by TUNEL staining according to the manufacturer’s instructions (*In Situ* Cell Death Detection Kit, POD, Roche, Germany). TUNEL-positive cells were analyzed and counted using ImageJ software. Apoptotic cells were counted in 2 (olfactory bulb), 10 (cerebrum), 3 (brain stem), or 2 fields (cerebellum) for each brain section per mouse at ×4 objective. Images are shown at ×10 objective.

### Statistical Analysis

Data are presented as means ± SD. The survival rates of the mice in different groups were analyzed using the Kaplan-Meier method and compared using the log-rank (Mantel-Cox) test. For comparisons between two groups, significance was analyzed by a *t*-test or Mann–Whitney *U* test depending on the normality of the data. For comparisons among three or more groups, significance was determined using a one-way ANOVA or Kruskal-Wallis ANOVA test depending on the normality of the data. All data were analyzed by GraphPad Prism software (version 6.01). *P* < 0.05 was considered significant.

## Results

### *Pb*YOP1 Deficiency Attenuates the Virulence of Parasites in ECM

**Figure 1:**
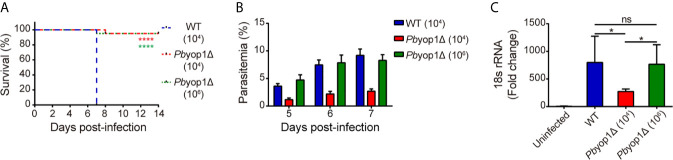
*Pb*YOP1 deficiency attenuates the virulence of parasites in inducing ECM. **(A)** Survival curve of C57BL/6 mice infected with WT (1×10^4^, n = 22) or *Pb*yop1Δ parasites (1×10^4^, n = 21; 1×10^6^, n = 20). Data are combined from three independent experiments. ****P < 0.0001 as determined by log-rank (Mantel-Cox) test. **(B)** 7 days post-infection, 1×10^6^ *Pb*yop1Δ-infected mice (n = 10) developed peripheral blood parasitemia similar to mice infected with 1×10^4^ WT parasites (n = 10). **(C)** Real-time PCR analysis of *P. berghei* 18S rRNA expression in the brain. Mouse β-actin was used as the internal control (n = 5/group). Data are presented as mean ± SD. *P < 0.05; ns, not significant as determined by Kruskal-Wallis ANOVA followed by Dunn’s multiple comparisons test.

To compare CM development by WT or *Pb*yop1Δ parasites and to eliminate the parasitemia-associated difference, C57BL/6 mice were intravenously inoculated with 1×10^4^ or a high infectious dose of 1×10^6^ *Pb*yop1Δ parasites, or with 1×10^4^ of WT parasites. As shown previously, all WT parasites-infected mice developed neurological symptoms classified to stage 4 or 5 (stage 4: reduced responsiveness to stimulation and/or ataxia and/or respiratory distress/hyperventilation; stage 5: prostration and/or paralysis and/or convulsions), thus counted as ECM-positive, and died within 6-8 dpi (11). In contrast, more than 90% of 1×10^6^ *Pb*yop1Δ parasites-infected mice did not display any stage 4/5 signs, thus considered ECM-negative, during 6-14 dpi (**Figure 1A**). Although the parasitemia was much lower in 1×10^4^ *Pb*yop1Δ parasites-infected mice, the ECM incidence was similar to that of 1×10^6^ *Pb*yop1Δ parasites-infected mice (**Figure 1B**). Therefore, in this study, the samples were acquired from *Pb*yop1Δ parasites-infected mice that did not suffer from CM and WT parasites-infected mice that developed CM.

Sequestration of iRBCs in microvasculature is responsible for disease severity in malaria (14). Parasite sequestration in the brain was determined based on quantification of *Pb*ANKA parasite-specific 18S rRNA by real-time PCR (12). At 7 dpi, the levels of parasite 18S rRNA in 1×10^6^ *Pb*yop1Δ-infected mice were at the same level as in WT-infected mice, both of which were significantly higher than in 1×10^4^ *Pb*yop1Δ-infected mice, consistent with their corresponding parasitemia (**Figure 1C**). These results indicate that *Pb*YOP1 plays a role in CM development but is dispensable for iRBC sequestration.

### *Pb*YOP1 Deficiency Does Not Affect T Cell Migration and Sequestration in Brain

As ECM is an immunopathological disease, the attenuated virulence of *Pb*yop1Δ parasites may be related to changes in immune response during *Plasmodium* infection. Numerous studies have demonstrated that CD8^+^ and CD4^+^ T cells respond to blood-stage *Plasmodium* parasite infection and are the principal effector cells involved in the pathogenesis of ECM (15–17). To investigate whether *Pb*YOP1 deficiency affects the T cell responses during ECM development, peripheral blood and brain mononuclear cells were isolated and quantified for the CD8^+^ and CD4^+^ T cell population by flow cytometry 7 dpi.

**Figure 2:**
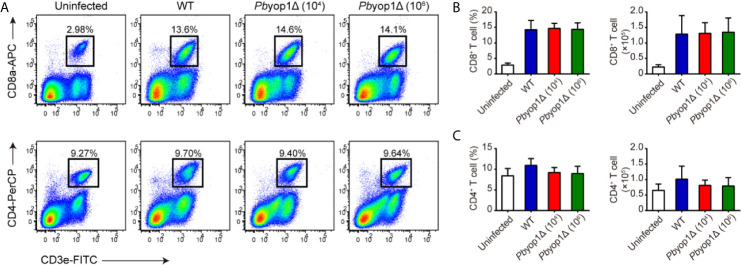
*Pb*yop1Δ parasites infection does not influence T cell response in peripheral blood. **(A)** Representative flow cytometry dot plots showing the CD8^+^ T cells and CD4^+^ T cells in peripheral blood mononuclear cells from uninfected, WT parasite-infected (10^4^), and *Pb*yop1Δ parasite-infected (10^4^ or 10^6^) mice 7 dpi. The frequency and cell number of CD8^+^ T cells **(B)** and CD4^+^ T cells **(C)** was quantified. Data are presented as mean ± SD (n = 6/group) and are representative of three independent experiments. Analyses were carried out by one-way ANOVA followed by Tukey’s multiple comparison test.

Circulating T cells are associated with the host systemic immune response to control the parasite burden and eradicate *Plasmodium* infection (18). As expected, CD8^+^ T cells in peripheral blood were significantly increased after infection, while no difference was detected among 1×10^4^ WT, 1×10^4^ *Pb*yop1Δ or 1×10^6^ *Pb*yop1Δ-infected mice (**Figures 2A, B**). Similarly, both the frequency and cell number of CD4^+^ T cell in peripheral blood did not change among the three infection schemes (**Figures 2A, C**). These results suggest that *Pb*yop1Δ parasite primes similar number of activated T cells as the WT parasite does.

**Figure 3:**
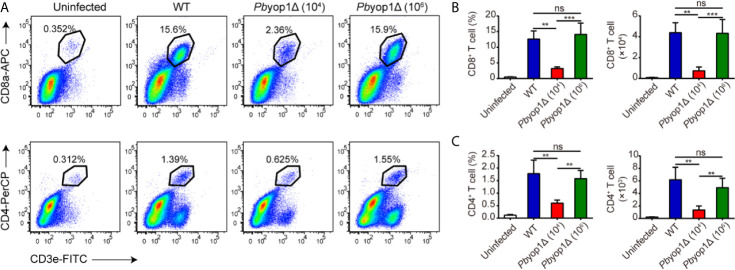
*Pb*yop1Δ parasites infection does not influence T cell infiltration in the brain. **(A)** Representative flow cytometry dot plots showing the frequency of CD8^+^ and CD4^+^ T cells sequestered in the brains of uninfected, WT parasites (10^4^)-infected, and *Pb*yop1Δ parasites (10^4^ and 10^6^)-infected mice 7 dpi. The frequency and number of CD8^+^ T cells **(B)** and CD4^+^ T cells **(C)** were quantified. Data are shown as mean ± SD (n = 5/group) and are representative of three independent experiments. **P < 0.01, ***P < 0.001; ns, not significant as determined by one-way ANOVA followed by Tukey’s multiple comparison test.

In the central nervous system, CD8^+^ and CD4^+^ T cells are sequestered in the microvasculature by adhering to the endothelial cells. Consistent with sequestration of iRBCs, CD8^+^ and CD4^+^ T cells were equivalently sequestered in the brain according to the levels of parasitemia in the three infection schemes. The proportion and number of CD8^+^ and CD4^+^ T cells were similar between WT and 1×10^6^ *Pb*yop1Δ-infected mice, and were both significantly higher than in 1×10^4^ *Pb*yop1Δ-infected mice (**Figure 3**). These results suggest that T cell sequestration in the brain was not affected by deletion of *Pb*YOP1.

CXCR3 is an important chemokine receptor associated with the migration of T cells into the brain and subsequent development of ECM (19, 20). To determine whether migration of T cell to brain was insufficient in *Pb*yop1Δ-infected mice, CXCR3 expression on CD8^+^ and CD4^+^ T cells in the peripheral blood and brain 7 dpi was measured by flow cytometry, and analyzed by the mean fluorescence intensity (MFI) and the frequency of CXCR3-positive T cells (**Supplementary Figures 1A–F**). In both the blood and brain, CXCR3 expression on CD8^+^ and CD4^+^ T cells was up-regulated upon infection, but no detectable difference was found between WT and *Pb*yop1Δ parasite-infected mice. Next, we examined the expression of CXCL9 and CXCL10 in brain, the CXCR3 ligands that facilitated peripheral CXCR3-positive T cells migrating up the chemokine gradient to the brain (20). Levels of CXCL9 and CXCL10 in brains were significantly reduced in 1×10^4^ *Pb*yop1Δ-infected mice, but no difference was observed between WT and 1×10^6^ *Pb*yop1Δ-infected mice (**Supplementary Figure 1G**). These results suggest that deletion of *Pb*YOP1 does not affect T cell migration.

### Th1 Cells Are Decreased in the Brains of *Pb*yop1Δ-Infected Mice

**Figure 4:**
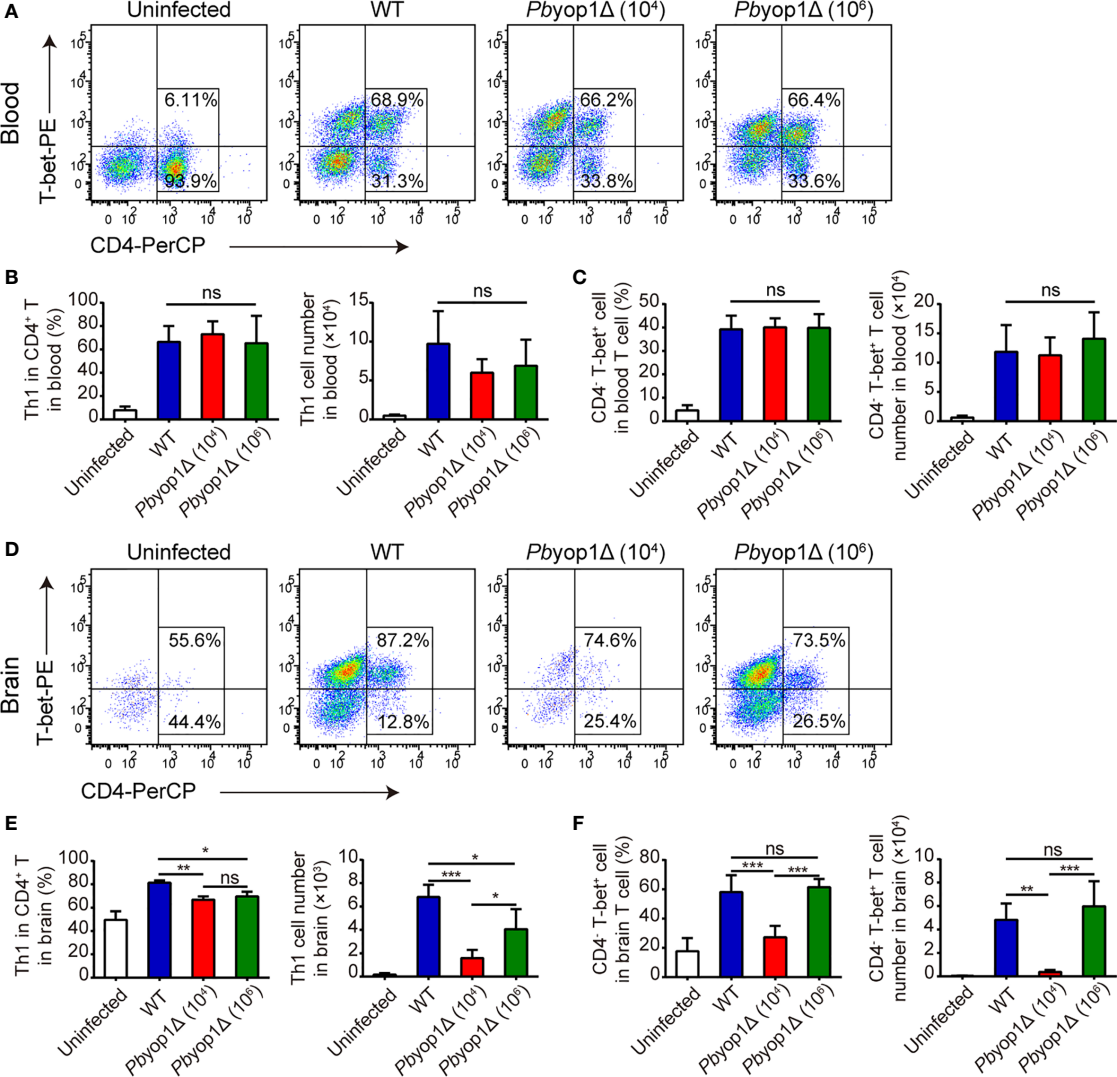
Th1 cells are decreased in the brains of *Pb*yop1Δ parasite-infected mice. **(A)** Representative flow cytometry dot plots showing Th1 cells in the peripheral blood of uninfected, WT parasites-infected (10^4^), and *Pb*yop1Δ parasites-infected (10^4^ or 10^6^) mice 7 dpi gated on CD3^+^ cells. **(B)** The frequency of Th1 cells in CD4^+^ T cells and the cell number of Th1 cells in peripheral blood were quantified. **(C)** The frequency of CD4^-^ T-bet^+^ cells in T cells and cell number of CD4^-^ T-bet^+^ T cells in peripheral blood were quantified. **(D)** Representative flow cytometry dot plots of Th1 cells in the brains of mice. **(E)** The frequency of Th1 cells in CD4^+^ T cells and the cell number of Th1 cells in brain were quantified. **(F)** The frequency of CD4^-^ T-bet^+^ cells in T cells and cell number of CD4^-^ T-bet^+^ T cells in brain were quantified. Data are displayed as mean ± SD (n = 6/group) and are representative of three independent experiments. *P < 0.05, **P < 0.01, ***P < 0.001; ns, not significant as determined by one-way ANOVA followed by Tukey’s multiple comparison test.

CD4^+^ T cells have the capacity to differentiate into one of several functionally distinct subsets. Th1 cells mediate the pro-inflammatory response and contribute to the development of ECM (21, 22). Because the total CD4^+^ T cell number in blood and brain was not different between WT and 1×10^6^ *Pb*yop1Δ-infected mice, we examined the levels of Th1 cells, defined as CD4^+^ T-bet^+^, in the induction of ECM. At 7 dpi, the percentage of Th1 cell in CD4^+^ T lymphocytes and the number of Th1 cells in peripheral blood were increased compared to the uninfected group, while there were no remarkable differences among the three infected groups (**Figures 4A, B**). The frequency of Th1 cells in the CD4^+^ T cell population was also increased in brains upon infection. However, the Th1 cell proportion in CD4^+^ T cell population of brain was significantly lower in *Pb*yop1Δ-infected group than in WT-infected group, and no changes were detected between 1×10^4^ and 1×10^6^ *Pb*yop1Δ-infected mice (**Figures 4D, E**). The absolute number of Th1 cells in brains was also lower in *Pb*yop1Δ-infected mice, particularly in the 1×10^4^ infected group (**Figure 4E**). Because T-bet is important for not only the differentiation of Th1 cells during induction of ECM, but also the generation of pathogenic CD8^+^ T cells (22), we calculated the frequencies and numbers of CD4^-^ T-bet^+^ T cells, most of which were likely CD8^+^ T-bet^+^ T cells, in peripheral blood and in brain (**Figures 4C, F** ), and observed no significant difference between WT and 1×10^6^ *Pb*yop1Δ-infected groups.

We also tested regulatory T cell (Treg), another CD4^+^ T cell subset that may play a regulatory role in preventing the induction of ECM and in controlling fatal pathogenesis (23, 24). No significant difference was detected between WT- and *Pb*yop1Δ-infected mice (**Supplementary Figure 2**). Taken together, these data suggest that *Pb*YOP1 deficiency in parasites downregulates Th1 cell sequestration in the brain while has no effect on Tregs.

### *Pb*YOP1 Deficiency Causes Reduced Secretion of IFN-γ and TNF-α

**Figure 5:**
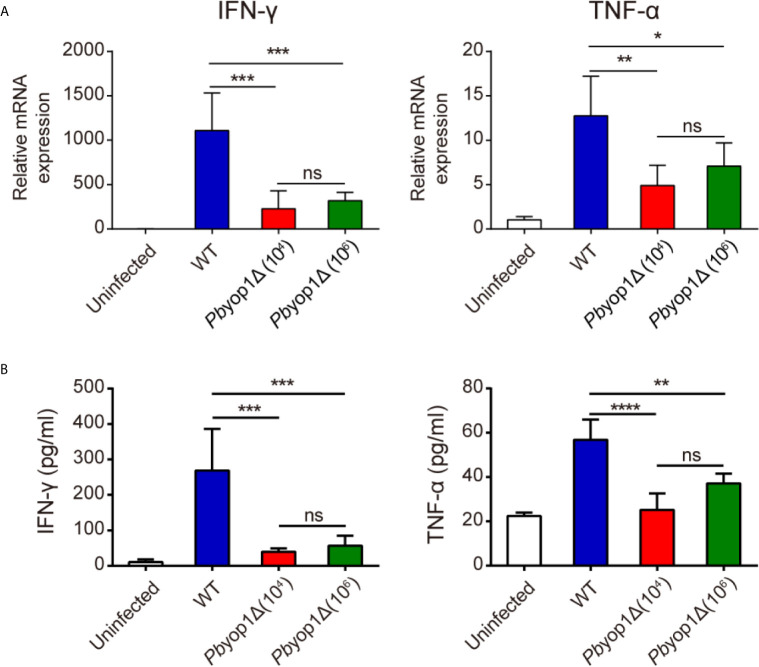
IFN-γ and TNF-α expression are decreased in *Pb*yop1Δ-infected mice. **(A)** IFN-γ and TNF-α mRNA expressions relative to β-actin in brain samples of uninfected and infected mice were evaluated by real-time PCR 7 dpi. **(B)** Serum IFN-γ and TNF-α levels were quantified by ELISA 7 dpi. Data are presented as mean ± SD (n = 5/group) and are representative of three independent experiments. *P < 0.05, **P < 0.01, ***P < 0.001, ****P < 0.0001; ns, not significant as determined by one-way ANOVA followed by Tukey’s multiple comparison test.

Th1 cell is responsible for the secretion of pro-inflammatory cytokines (25, 26), which are important in activating other immune cells to respond to infection and in the pathogenesis of ECM (27). Since Th1 cells were reduced in the brains of *Pb*yop1Δ-infected mice during ECM construction, we examined whether deletion of *Pb*yop1 would influence the production of pro-inflammatory cytokines IFN-γ and TNF-α, two crucial Th1-type cytokines. IFN-γ is required to activate brain endothelial cells and participate in brain endothelial cells cross-presentation of parasite antigen (28). Although TNF-α plays a dispensable role in ECM development, it exacerbates cerebral pathology (29, 30). At 7 dpi, the mRNA expression of IFN-γ and TNF-α were significantly decreased in the brains of *Pb*yop1Δ-infected mice compared to WT-infected mice (**Figure 5A**). The levels of IFN-γ and TNF-α in serum displayed similar results (**Figure 5B**). These results suggest that reduction of brain-trapped Th1 cells and subsequent reduction of IFN-γ and TNF-α may attribute to the blockage of CM development in *Pb*yop1Δ-infected mice.

### *Pb*YOP1 Deficiency Causes Reduced Cell Adhesion in the Brainstem

Under inflammatory conditions during *Plasmodium* infection, pro-inflammatory cytokines such as IFN-γ induce brain endothelial activation and local inflammation (31). Activation of brain endothelial cells is associated with leukocyte adhesion, parasite sequestration, and function of antigen cross-presentation (28, 32). A hallmark of endothelial activation is the upregulated expression of adhesion molecules, such as ICAM-1, VCAM-1, and CD36, on the endothelium of cerebral microvessels (33, 34). At 7 dpi, the mRNA expressions of ICAM-1 and VCAM-1 were upregulated in the brain, but no prominent differences were detected among the three infected schemes. CD36 transcripts were more abundant in brains of 1×10^6^ *Pb*yop1Δ-infected mice (**Supplementary Figure 3**). The elevation of CD36 is likely mostly contributed by the induced expression in innate immune cells in brain, because CD36 is also a scavenger receptor employed by phagocytes like monocytes, macrophages and microglia. Higher initial infection dose of parasites would trigger a more intensive innate immune response for phagocytic clearance of iRBCs (35). It is also reasonable to speculate that sustained CD36 expression in brains of 1×10^6^ *Pb*yop1Δ-infected mice reflects a continuous demand on innate immunity due to attenuation in adaptive immune response.

**Figure 6:**
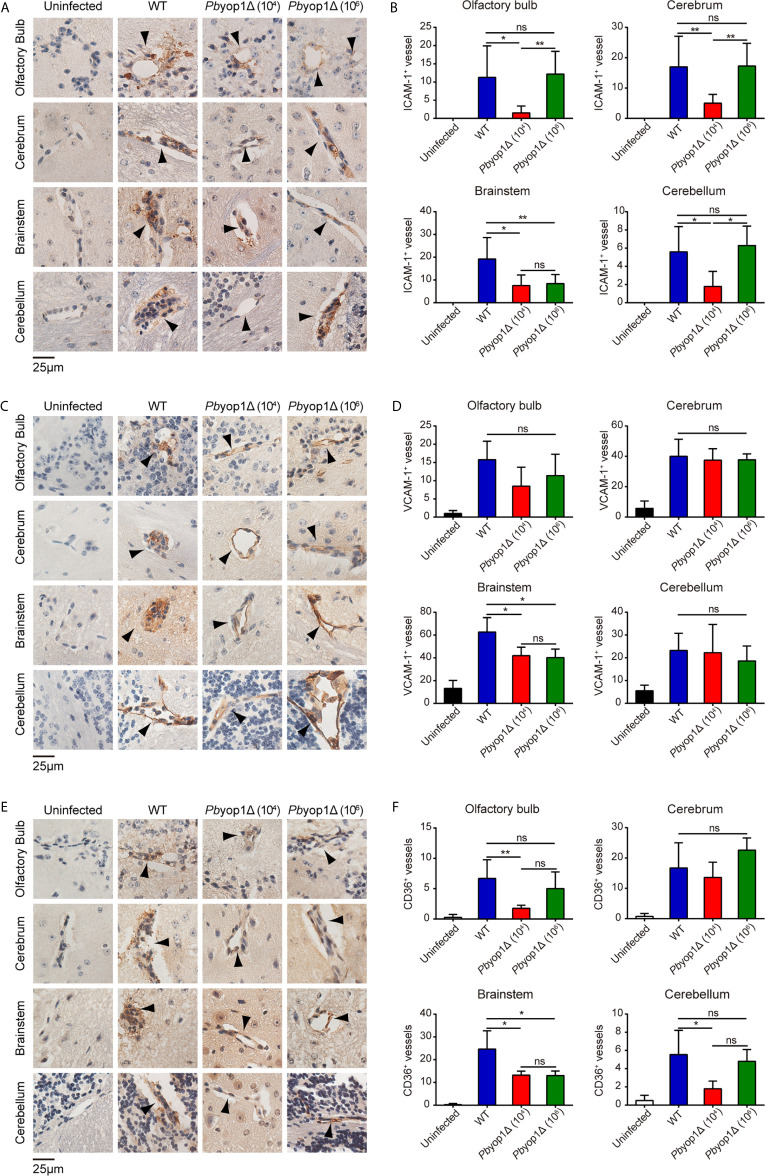
ICAM-1, VCAM-1, and CD36 expression are downregulated in the brainstem of *Pb*yop1Δ parasites-infected mice. **(A, C, E)** Representative images of IHC staining of ICAM-1, VCAM-1, or CD36 in different brain regions of mice infected with 10^4^ WT parasites (n = 11), 10^4^ *Pb*yop1Δ parasites (n = 5), or 10^6^ *Pb*yop1Δ parasites (n = 5) and uninfected mice (n = 4). **(B, D, F)** The bar graphs show quantification of the data in **(A, C, E)**. ICAM-1, VCAM-1, or CD36-positive vessels (black arrows) were quantified for each sagittal brain section in 6 fields (olfactory bulb), 20 fields (cerebrum), 15 fields (brainstem), and 5 fields (cerebellum); one brain section per mouse. Data are presented as mean ± SD. Differences among the three groups were analyzed using Kruskal-Wallis ANOVA followed by Dunn’s multiple comparisons test: *P < 0.05, **P < 0.01; ns, not significant.

Fatal ECM is frequently linked to severe brainstem pathology (36). To test whether the extent of endothelial activation was distinct in specific brain regions of infected mice, we examined the protein levels of ICAM-1, VCAM-1, and CD36 by IHC staining and counting stain-positive vessels in multiple brain regions, including olfactory bulb, cerebrum, brainstem and cerebellum. At 7 dpi, the numbers of ICAM-1^+^, VCAM-1^+^ and CD36^+^ vessels were significantly lower in the brainstems of *Pb*yop1Δ-infected mice compared to WT-infected mice (**Figure 6**). Variation in ICAM-1^+^, VCAM-1^+^, and CD36^+^ vessels in other brain regions were also compared among the three infected schemes, but the differences were not as significant as detected in the brainstem. These results indicate insufficient endothelial activation in the brainstem in *Pb*yop1Δ-infected mice, which may influence the subsequent immune pathologies mediated by CD8^+^ T cells.

### Cell Apoptosis Is Attenuated in the Brain of *Pb*yop1Δ-Infected Mice

During ECM pathogenesis, the activated endothelial cells adhere CD8^+^ T cells and present parasite-specific antigens to T cells. The effector CD8^+^ T cells secrete granzyme B and perforin to induce damage of intercellular tight junctions of the endothelium, trigger apoptosis of endothelial cells and neuronal cells, which disrupting the BBB and finally impairing central nervous system function (36–38). Thus, we measured the expression of granzyme B and perforin in the brain 7 dpi. The granzyme B and perforin mRNA levels were significantly decreased in the brains of *Pb*yop1Δ-infected mice compared to WT-infected mice (**Supplementary Figure 4**).

**Figure 7:**
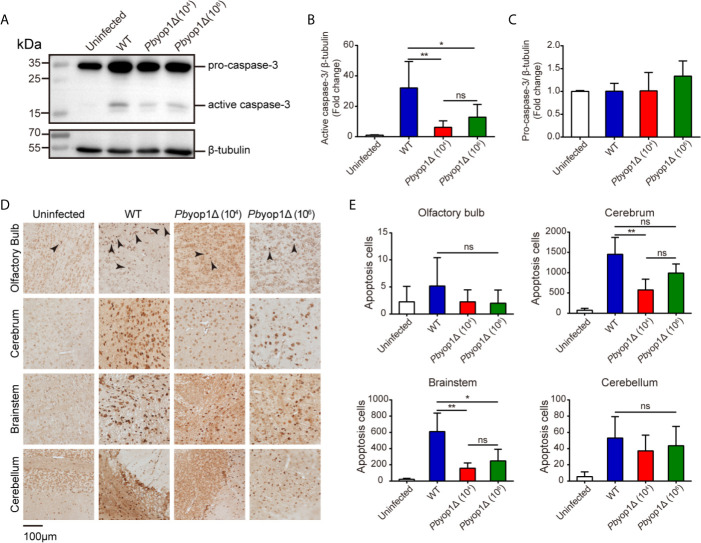
Cell apoptosis is attenuated in the brains of *Pb*yop1Δ parasite-infected mice. **(A)** Representative image of caspase-3 expression in the brains of uninfected (n = 4), WT parasites-infected (10^4^, n = 11), and *Pb*yop1Δ parasites-infected (10^4^ or 10^6^, n = 5) mice 7 dpi. The bar graphs show the quantification of the data in (A). The gray value of active caspase-3 **(B) **and pro-caspase-3 **(C)** is normalized to β-tubulin. **(D)** Representative images of TUNEL staining of apoptotic cells in different brain regions 7 dpi. **(E)** Apoptotic cells shown in **(D)** were quantified for each sagittal brain section in 2 fields (olfactory bulb), 10 fields (cerebrum), 3 fields (brainstem), and 2 fields (cerebellum); one brain section per mouse. Data are presented as the mean ± SD. Differences among the three groups were analyzed using Kruskal-Wallis ANOVA followed by Dunn’s multiple comparisons test: *P < 0.05, **P < 0.01; ns, not significant.

Caspase-3 is the main executioner of apoptosis and activated during ECM (39). Granzyme B can directly cleave pro-caspase-3, resulting in an active caspase-3. Activated caspase-3 induces DNA fragmentation and cell death, leading to the lethal pathogenesis of ECM (40, 41). To further examine the brain cell damage, we tested the activation of caspase-3 and cell apoptosis in ECM. At 7 dpi, active caspase-3 was significantly decreased in the brains of *Pb*yop1Δ-infected mice compared to WT-infected mice. Consistent with ECM incidence, no significant difference was detected between 1×10^4^ and 1×10^6^ *Pb*yop1Δ-infected mice (**Figures 7A, B**). In addition, the pro-caspase-3 protein levels did not change after infection (**Figures 7A, C**).

Cell apoptosis was also detected *in situ* by TUNEL staining. Apoptotic cells were counted in the four brain regions. *Pb*yop1Δ parasite infection induced cell death was significantly reduced in the brainstem versus WT parasite infection (**Figures 7D, E**). Cell death was also decreased in the olfactory bulb, cerebrum, and cerebellum, where it was mild in *Pb*yop1Δ-infected mice. Cell apoptosis was consistent with endothelial activation in different brain regions. These results suggest that CD8^+^ T cell-mediated intracerebral cell apoptosis is attenuated in *Pb*yop1Δ parasite infection.

### Inflammation Is Further Alleviated in the Brains of *Pb*yop1Δ Parasite-Infected Mice

**Figure 8:**
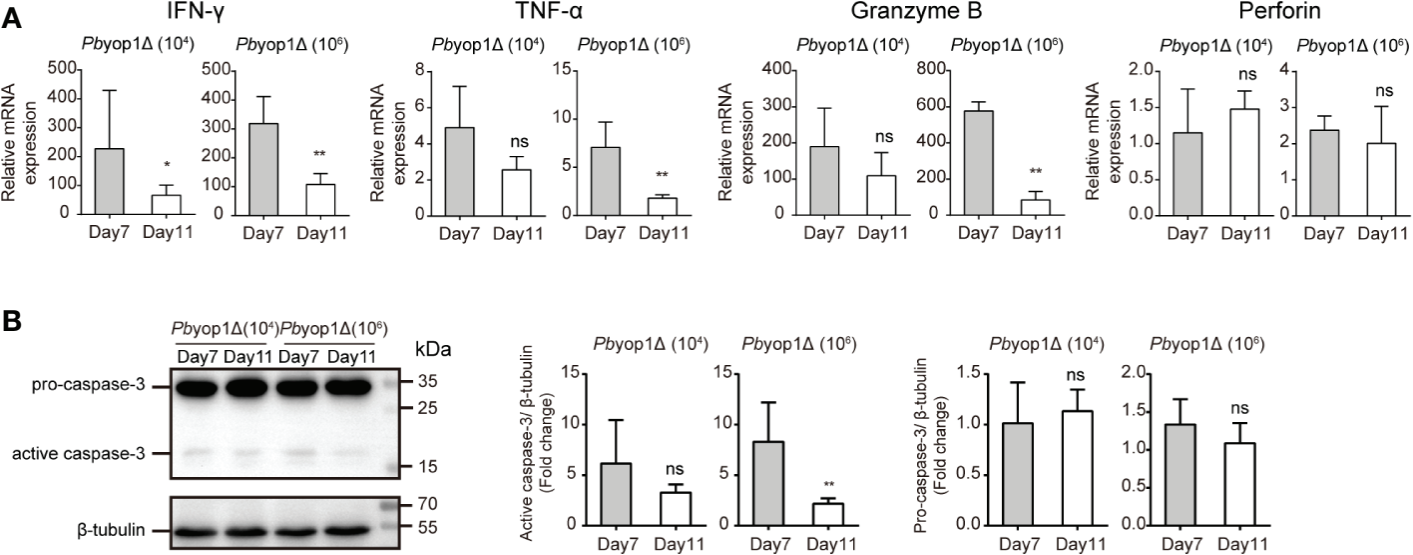
Inflammation is further alleviated in the brains of *Pb*yop1Δ parasite-infected mice. **(A)** IFN-γ, TNF-α, granzyme B, and perforin mRNA expression relative to β-actin in brains from mice infected with 10^4^ or 10^6^ *Pb*yop1Δ parasites was evaluated by real-time PCR 7 and 11 dpi. **(B)** Representative image and quantification of caspase-3 expression in the brain 7 and 11 dpi. Data are presented as mean ± SD (n = 5/group) and are representative of three independent experiments. *P < 0.05, **P < 0.01; ns, not significant as determined by the Mann-Whitney *U* test.

Because the mice infected with *Pb*yop1Δ parasites died of severe anemia without distinct manifestations of ECM more than 3 weeks post-infection, we tested the expression of cytokines and cytolytic molecules after the time frame for the onset of ECM, 11 dpi (42). The expression of IFN-γ, TNF-α, granzyme B, and perforin (**Figure 8A**), and the activation of apoptosis molecule caspase-3 (**Figure 8B**) were down-regulated in the brains of *Pb*yop1Δ parasite-infected mice 11 dpi compared to 7 dpi, particularly in 1×10^6^ *Pb*yop1Δ-infected mice. However, the cell death in the brain neither expanded nor recovered for the irreversibility of apoptosis 11 dpi (data not shown). These data imply that *Pb*yop1Δ parasites not only give rise to mild pro-inflammatory responses and cytotoxic effects of brain infiltrating T cells that were not sufficient to lead to lethal pathogenesis of brain during ECM induction, but also that these immunopathological changes decreased gradually.

## Discussion

YOP1 in *P. berghei* ANKA is the homolog of DP1/REEP5 in humans or Yop1p in *Saccharomyces cerevisiae*. It is one of the integral membrane proteins that generate ER tubules by inducing high curvature in the membrane (8). *Pb*YOP1-deleted *P. berghei* parasites were generated to explore the function of this important ER tubule-shaping protein in the *Plasmodium* parasite and malaria. We found that *Pb*YOP1 had a profound effect on the parasite growth rate and the pathogenesis of ECM in blood-stage infection. As described recently, the slow growth rate of parasites in erythrocytic stage is associated with a dysfunction of hemoglobin degradation in the digestive vacuole and disordered parasite metabolism (11). However, the mechanism of susceptible mice infected with *Pb*yop1Δ parasites surviving from ECM induction is unclear. It is reported that the T cell response to malaria may contribute to ECM (43, 44). In this study, we analyzed the effect of *Pb*YOP1 on parasite virulence by detecting the T cell response associated with the pathogenesis of ECM.

ECM is a complex neurological syndrome. In previous studies, iRBC sequestration in the brain microvasculature was associated with the development of ECM (45, 46). Although the growth rate of *Pb*yop1Δ parasites is significantly decreased in the asexual phase, parasite sequestration in the brains of 10^6^ *Pb*yop1Δ parasites-infected mice is comparable to that of 10^4^ WT parasites-infected mice and fails to induce ECM. Sequestration of iRBCs only is inadequate to induce the brain injury leading to the fatal syndrome during infection.

Both CD4^+^ and CD8^+^ T cells have been shown to contribute to ECM development (47, 48). The mechanisms by which CD4^+^ T cells mediate cerebral complications have not been fully elucidated, but it is thought to involve the production of Th1-type cytokines, such as IFN-γ, that exacerbate the inflammatory cascade responsible for local and systemic inflammation in cerebral malaria (23). CD8^+^ T cell depletion or ablation of effective functions completely abrogates the development of ECM (27, 49). Brain infiltrating CD8^+^ T cells induce opening of endothelium tight junction, endothelial cell apoptosis and other intracerebral cell apoptosis in a granzyme B and perforin-dependent manner (50, 51). In addition, perforin secreted by CD8^+^ T cells is sufficient to cause cell death, disrupted BBB, and fatal brain edema in the specific regions of the brain, including brainstem and olfactory bulb, during ECM (52), indicating that the killing effect mediated by CD8^+^ T cells plays a vital and precise role in ECM pathology. Upon examination of sequestered T cells during infection, the *Pb*YOP1-deficient parasites had attenuated virulence without influencing sequestration of the total CD4^+^ and CD8^+^ T cells in the brain, and the frequency and cell number were comparable between WT and 10^6^ *Pb*yop1Δ-infected mice. Additionally, expression of CXCR3, which is associated with T cell migration, did not change in the three infection schemes including 10^4^ *Pb*yop1Δ-infected mice.

Some studies have demonstrated that the pro-inflammatory Th1 response is involved in the pathogenesis of ECM (22, 42). Consistent with the morbidity of ECM, the sequestration of Th1 cells in the brain was significantly decreased in *Pb*yop1Δ parasite-infected mice. Stimulation of T cell receptor and other extrinsic factors, particularly cytokines, which are associated with STAT activation, are crucial for the appropriate differentiation of CD4^+^ T cell subsets (53, 54). Although the number of Th1 cells in peripheral blood did not change in *Pb*yop1Δ-infected mice compared to WT-infected mice, Th1 cells sequestered in brain decreased remarkably. This may due to the attenuated virulence of *Pb*yop1Δ parasites, which may influence parasites and leukocytes sequestrations in brain microvasculature, the process of parasite-derived antigen cross-presentation in endothelial cells, and the pro-inflammatory response inducing differentiation of CD4^+^ T cells (55).

>The cytokines associated with the pathogenesis of ECM were also detected in this study. The expression of pro-inflammatory cytokines IFN-γ and TNF-α (56, 57) and cytotoxic molecules granzyme B and perforin (51, 58), and the activation of the main executioner of apoptosis caspase-3 (59) were significantly reduced in *Pb*yop1Δ parasites-infected mice; all of these reductions directly protect the *Pb*yop1Δ parasite-infected mice from ECM. Moreover, ECM occurs 6-10 dpi (42), and these pro-inflammatory factors were further decreased 11 dpi in *Pb*yop1Δ-infected mice; thus, the inflammation induced by the *Pb*yop1Δ parasite is not only attenuated, but also down-regulated gradually.

The brainstem regulates many vital functions, such as the cardiovascular and respiratory systems, and it is likely that mice succumb to ECM due to the widespread inflammation and neuron death observed in this brain region (36, 60). The pathogenesis in the brainstem was significantly alleviated in *Pb*yop1Δ parasites infection. We detected that the adhesion molecules expressed on microvessels were decreased in the brainstem, as measured by IHC staining of ICAM-1, VCAM-I, and CD36. *Pb*yop1Δ parasite infection induced cell apoptosis detected by *in situ* TUNEL staining was also reduced in the four brain regions (olfactory bulb, cerebrum, brainstem, and cerebellum), particularly in the brainstem, consistent with the IHC results. While the connection of ICAM-1 and VCAM-1 to CM development is straightforward, the case for CD36 is complicated. CD36 plays a dual role in malaria: its expression in phagocytes induced at early stage of infection has an important effect on parasites clearance (61); whereas that in endothelial cells mediates parasite sequestration in microvasculature of organs (62). Notably, murine CD36-mediated sequestration is not essential for CM pathology (62). However, it has been reported that ICAM-1 and CD36 synergize to mediate cytoadherence of *Plasmodium falciparum*-infected RBCs to human endothelial cells (63), suggesting that CD36 might contribute to CM pathogenesis in a collaborative manner.

After invading the erythrocyte, hundreds of proteins are exported out of the parasite and beyond the parasitophorous vacuole membrane to numerous locations within the parasite-infected erythrocyte (64). *Pb*YOP1 deficiency may affect the ER function and tubule formation, resulting in disordered parasite metabolism and a defect in protein secretion. The pathologies in ECM are initially induced by the parasite antigen presented on the activated endothelial cell MHC I molecule and recognized by the specific T cell receptor on CD8^+^ T cells (16, 65). Comparative analysis between the secretome of WT and *Pb*YOP1-deleted parasites would reveal vital clues for understanding the development of CM.

The *Pb*YOP1-deleted parasites offer a unique and important opportunity for further understanding of ECM. Specifically, previous studies showed that in non-ECM *Pb* NK65-infected C57BL/6 mice, iRBCs were not efficiently accumulated in brain microvessels (4). These differences between known non-ECM parasites and the *Pb*YOP1-deleted parasites suggest that the *Pb*yop1Δ parasites could move at least one step further in ECM pathogenesis when compared to existing non-ECM *Plasmodium* strains, and thus become more useful materials for pinpointing the key elements during ECM development.

## Data Availability Statement

The original contributions presented in the study are included in the article/**Supplementary Material**. Further inquiries can be directed to the corresponding authors.

## Ethics Statement

The animal study was reviewed and approved by Institutional Animal Care and Use Committee (IACUC) of Tianjin Medical University.

## Author Contributions

QW and XS designed the study. LH and XS performed the experiments. LH analyzed the data. LH, XS and QW wrote the paper. All authors contributed to the article and approved the submitted version.

## Funding

This work was supported by the National Natural Science Foundation of China (grant number 32070701 to QW), the Startup Funds from Tianjin Medical University (grant numbers 115004/000012 and 11601502/DW0114 to QW), the Science & Technology Development Fund of Tianjin Education Commission for Higher Education (grant number 2016KJ0138 to QW and 2018KJ085 to XS), and the National Laboratory of Biomacromolecules (grant number 2018kf05 and 2020kf11 to QW).

## Conflict of Interest

The authors declare that the research was conducted in the absence of any commercial or financial relationships that could be construed as a potential conflict of interest.

## Supplementary Material

Supplementary Figure 1
*Pb*YOP1 deficiency in parasites does not affect T cell migration to brain. **(A)** Representative histograms showing CXCR3 expression on CD8^+^ and CD4^+^ T cells in peripheral blood from uninfected, WT parasites (10^4^) -infected, and *Pb*yop1Δ parasites (10^4^ and 10^6^) -infected mice 7 dpi. **(B)** The frequency of CXCR3^+^ CD8^+^ T cells and CXCR3^+^ CD4^+^ T cells in blood. **(C)** The MFI of CXCR3 in CD8^+^ and CD4^+^ T cells. **(D–F)** Representative histograms **(D)** and quantification of CXCR3 expression in CD8^+^ and CD4^+^ T cells in the brain **(E, F)**. **(G)** mRNA expressions of CXCL9 and CXCL10 in the brain were measured by real-time PCR. Data are presented as mean ± SD (n = 6/group) and are representative of three independent experiments. **P* < 0.05, ***P* < 0.01; ns, not significant as determined by one-way ANOVA followed by Tukey’s multiple comparison test.

Supplementary Figure 2
*Pb*yop1Δ parasite has no effect on regulatory T cell accumulation in the blood and brain. **(A, B)** Representative flow cytometry dot plots showing regulatory T cells in the blood **(A)** and brain **(B)** of uninfected, WT parasites-infected (10^4^), and *Pb*yop1Δ parasites-infected (10^4^ or 10^6^) mice 7 dpi gated on CD4^+^ T cells. **(C, D)** The frequency and cell number of Tregs in total CD4^+^ T cells in the blood **(C)** or brains **(D)**. Data are presented as mean ± SD (n = 6/group) and are representative of three independent experiments. **P* < 0.05, ***P* < 0.01; ns, not significant as determined by one-way ANOVA followed by Tukey’s multiple comparison test.

Supplementary Figure 3 mRNA expression of adhesion molecule ICAM-1, VCAM-1 and CD36 in the brain. mRNA expressions of ICAM-1, VCAM-1, and CD36 relative to β-actin in brain samples from uninfected and infected mice were evaluated by real-time PCR 7 dpi. Data are presented as mean ± SD (n = 5/group) and are representative of three independent experiments. ****P* < 0.001; ns, not significant as determined by one-way ANOVA followed by Tukey’s multiple comparison test.

Supplementary Figure 4 Expression of granzyme B and perforin is decreased in the brains of *Pb*yop1Δ parasites-infected mice. Granzyme B and perforin mRNA expressions relative to β-actin in brain samples from uninfected and infected mice were evaluated by real-time PCR 7 dpi. Data are presented as mean ± SD (n = 5/group) and are representative of three independent experiments. **P* < 0.05, ***P* < 0.01, ****P* < 0.001; ns, not significant as determined by one-way ANOVA followed by Tukey’s multiple comparison test.
