# Supplementary figures and images for: Attenuated T Cell Responses Are Associated With the Blockade of Cerebral Malaria Development by YOP1-Deficient Plasmodium berghei ANKA
Source: Front Immunol. 2021 May 6;12:642585. doi: 10.3389/fimmu.2021.642585 (PMC8134684; doi:10.3389/fimmu.2021.642585)

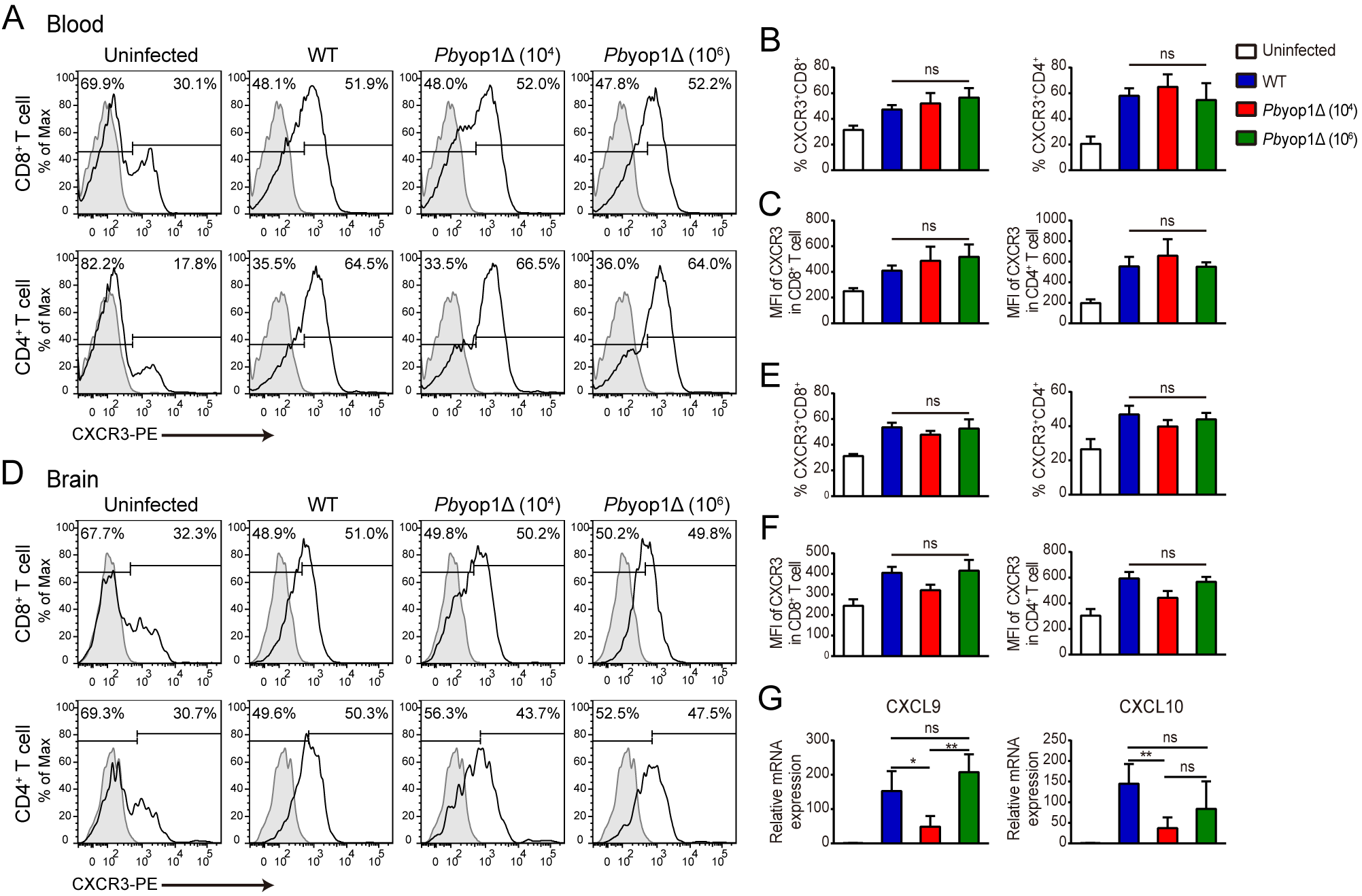

Supplement: Supplementary Figure 1 — PbYOP1 deficiency in parasites does not affect T cell migration to brain. (A) Representative histograms showing CXCR3 expression on CD8+ and CD4+ T cells in peripheral blood from uninfected, WT parasites (104) -infected, and Pbyop1Δ parasites (104 and 106) -infected mice 7 dpi. (B) The frequency of CXCR3+ CD8+ T cells and CXCR3+ CD4+ T cells in blood. (C) The MFI of CXCR3 in CD8+ and CD4+ T cells. (D–F) Representative histograms (D) and quantification of CXCR3 expression in CD8+ and CD4+ T cells in the brain (E, F). (G) mRNA expressions of CXCL9 and CXCL10 in the brain were measured by real-time PCR. Data are presented as mean ± SD (n = 6/group) and are representative of three independent experiments. *P < 0.05, **P < 0.01; ns, not significant as determined by one-way ANOVA followed by Tukey’s multiple comparison test. [file Image_1.tif]

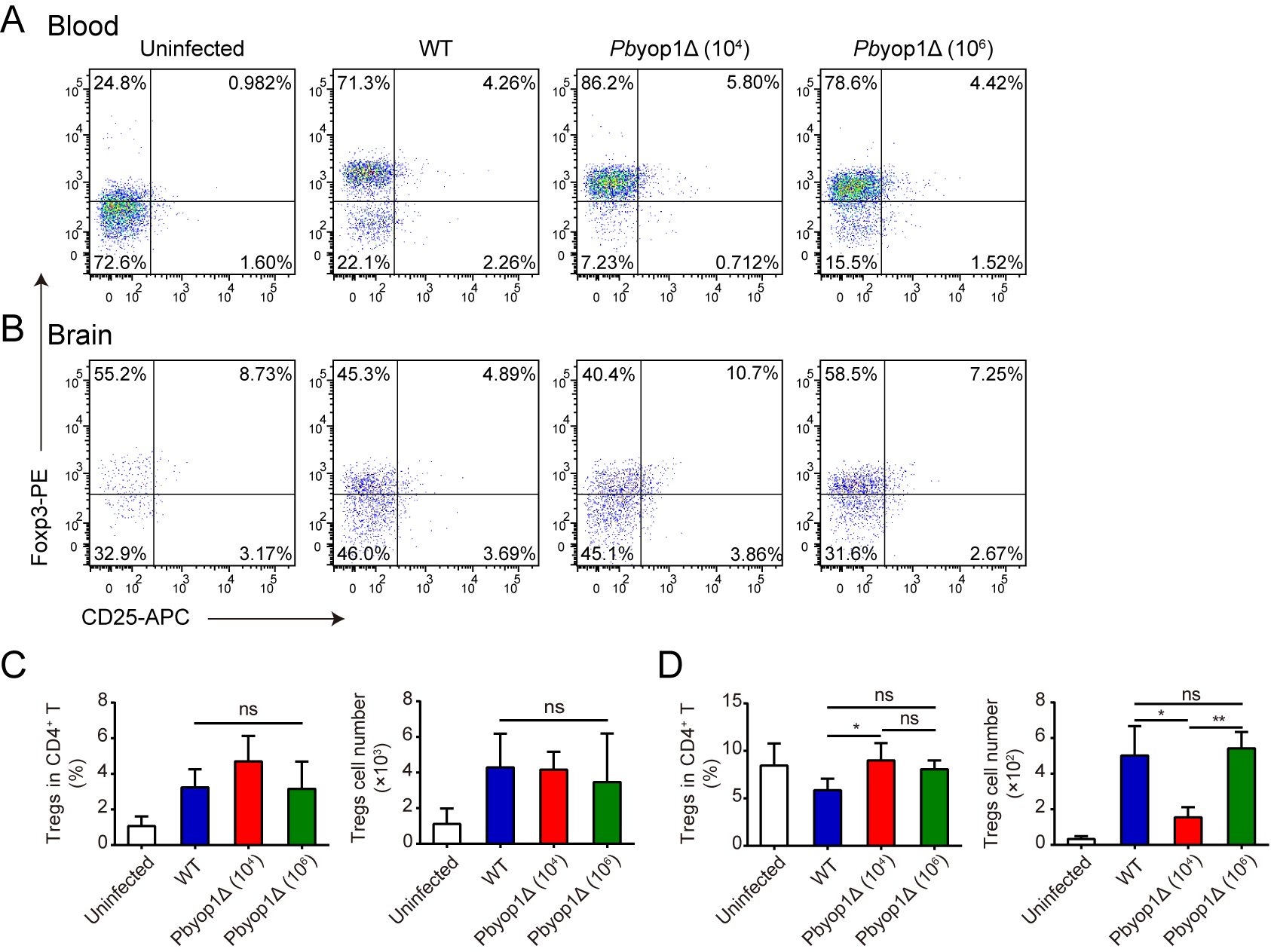

Supplement: Supplementary Figure 2 — Pbyop1Δ parasite has no effect on regulatory T cell accumulation in the blood and brain. (A, B) Representative flow cytometry dot plots showing regulatory T cells in the blood (A) and brain (B) of uninfected, WT parasites-infected (104), and Pbyop1Δ parasites-infected (104 or 106) mice 7 dpi gated on CD4+ T cells. (C, D) The frequency and cell number of Tregs in total CD4+ T cells in the blood (C) or brains (D). Data are presented as mean ± SD (n = 6/group) and are representative of three independent experiments. *P < 0.05, **P < 0.01; ns, not significant as determined by one-way ANOVA followed by Tukey’s multiple comparison test. [file Image_2.tif]

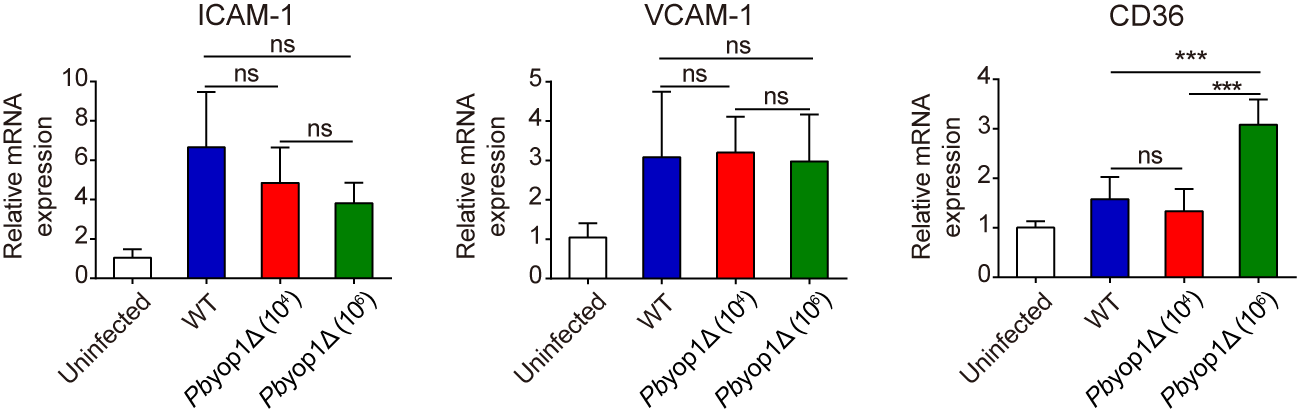

Supplement: Supplementary Figure 3 — mRNA expression of adhesion molecule ICAM-1, VCAM-1 and CD36 in the brain. mRNA expressions of ICAM-1, VCAM-1, and CD36 relative to β-actin in brain samples from uninfected and infected mice were evaluated by real-time PCR 7 dpi. Data are presented as mean ± SD (n = 5/group) and are representative of three independent experiments. ***P < 0.001; ns, not significant as determined by one-way ANOVA followed by Tukey’s multiple comparison test. [file Image_3.tif]

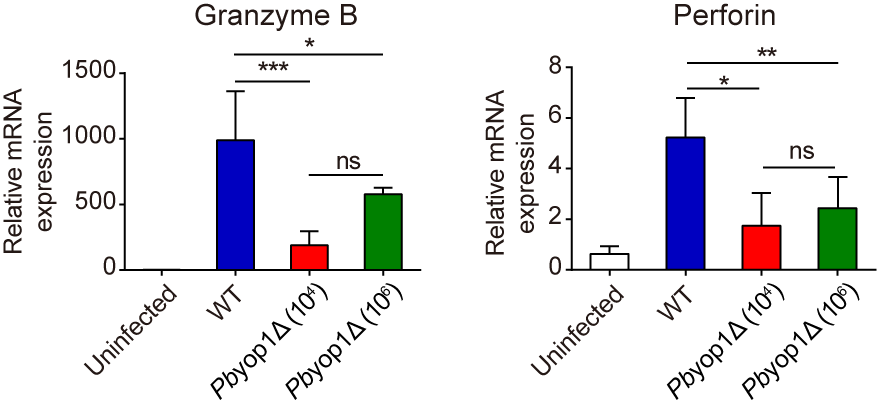

Supplement: Supplementary Figure 4 — Expression of granzyme B and perforin is decreased in the brains of Pbyop1Δ parasites-infected mice. Granzyme B and perforin mRNA expressions relative to β-actin in brain samples from uninfected and infected mice were evaluated by real-time PCR 7 dpi. Data are presented as mean ± SD (n = 5/group) and are representative of three independent experiments. *P < 0.05, **P < 0.01, ***P < 0.001; ns, not significant as determined by one-way ANOVA followed by Tukey’s multiple comparison test. [file Image_4.tif]
