# Supplementary material for: Attenuated T Cell Responses Are Associated With the Blockade of Cerebral Malaria Development by YOP1-Deficient Plasmodium berghei ANKA
Source: Front Immunol. 2021 May 6;12:642585. doi: 10.3389/fimmu.2021.642585 (PMC8134684; doi:10.3389/fimmu.2021.642585)
Supplement: Supplementary file 5 [file Table_1.docx]

**Supplementary Tables**

**Supplemental Table 1. Primers used in real-time PCR**

| Primer | Sequence |
| --- | --- |
| β-actin F | GGCTGTATTCCCCTCCATCG |
| β-actin R | CCAGTTGGTAACAATGCCATGT |
| *Pb* 18S rRNA F | AAGCATTAAATAAAGCGAATACATCCTTAC |
| *Pb* 18S rRNA R | GGAGATTGGTTTTGACGTTTATGTG |
| IFN-γ F | ATGAACGCTACACACTGCATC |
| IFN-γ R | CCATCCTTTTGCCAGTTCCTC |
| TNF-α F | CCCTCACACTCAGATCATCTTCT |
| TNF-α R | TGCTACGACGTGGGCTACAG |
| CXCL9 F | GCCATGAAGTCCGCTGTTCT |
| CXCL9 R | GGGTTCCTCGAACTCCACACT |
| CXCL10 F | GACGGTCCGCTGCAACTG |
| CXCL10 R | GCTTCCCTATGGCCCTCATT |
| ICAM-1 F | GAGATCACATTCACGGTGCTG |
| ICAM-1 R | AGCTGGAAGATCGAAAGTCCG |
| VCAM-1 F | GACATCTACTCTTTCCCCAAGG |
| VCAM-1 R | TGTTCATGAGCTGGTCACCCT |
| CD36 F | TTCTTCACAGCTGCCTTCTGA |
| CD36 R | ACGTGGCCCGGTTCTAATTCA |
| Granzyme B F | TCGACCCTACATGGCCTTAC |
| Granzyme B R | TGGGGAATGCATTTTACCAT |
| Perforin F | TTGGCCCATTTGGTGGTAAG |
| Perforin R | AGTCTCCCCACAGATGTTCTGC |
